# A Blade-Type Triboelectric-Electromagnetic Hybrid Generator with Double Frequency Up-Conversion Mechanism for Harvesting Breeze Wind Energy

**DOI:** 10.1021/acsami.4c04377

**Published:** 2024-06-21

**Authors:** Na Yang, Yingxuan Li, Zhenlong Xu, Yongkang Zhu, Qingkai He, Ziyi Wang, Xueting Zhang, Jingbiao Liu, Chaoran Liu, Yun Wang, Maoying Zhou, Tinghai Cheng, Zhong Lin Wang

**Affiliations:** † School of Mechanical Engineering, Hangzhou Dianzi University, Hangzhou 310018, China; ‡ Ministry of Education Engineering Research Center of Smart Microsensors and Microsystems, College of Electronics and Information, Hangzhou Dianzi University, Hangzhou 310018, China; § Beijing Institute of Nanoenergy and Nanosystems, Chinese Academy of Sciences, Beijing 101400, China

**Keywords:** triboelectric-electromagnetic hybrid generator, breeze wind energy harvesting, double frequency up-conversion, self-powered wireless sensor, energy management circuit, intelligent agriculture

## Abstract

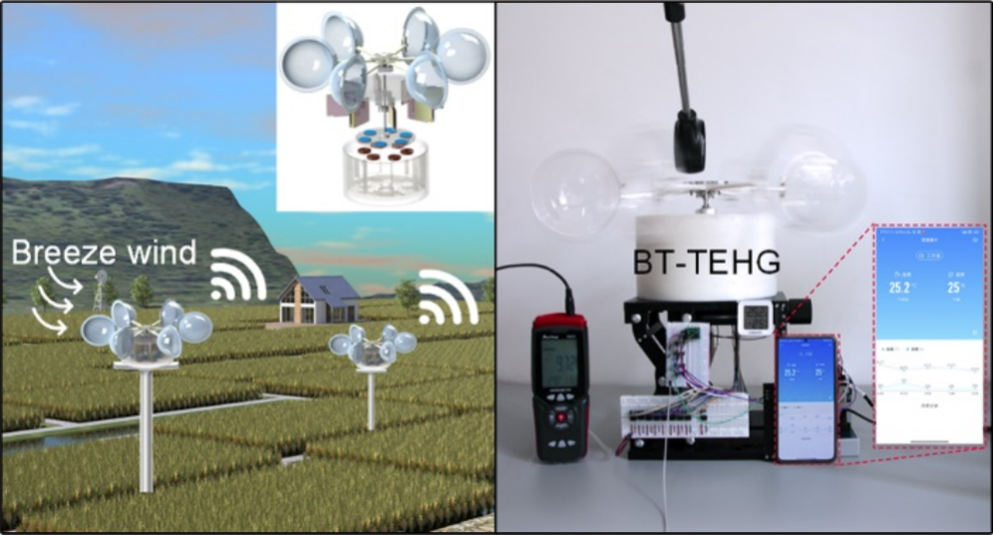

Triboelectric nanogenerators (TENGs) have garnered substantial attention in breeze wind energy harvesting. However, how to improve the output performance and reduce friction and wear remain challenging. To this end, a blade-type triboelectric-electromagnetic hybrid generator (BT-TEHG) with a double frequency up-conversion (DFUC) mechanism is proposed. The DFUC mechanism enables the TENG to output a high-frequency response that is 15.9 to 300 times higher than the excitation frequency of 10 to 200 rpm. Coupled with the collisions between tribomaterials, a higher surface charge density and better generating performance are achieved. The magnetization direction and dimensional parameters of the BT-TEHG were optimized, and its generating characteristics under varying rotational speeds and electrical boundary conditions were studied. At wind speeds of 2.2 and 10 m/s, the BT-TEHG can generate, respectively, power of 1.30 and 19.01 mW. Further experimentation demonstrates its capacity to charge capacitors, light up light emitting diodes (LEDs), and power wireless temperature and humidity sensors. The demonstrations show that the BT-TEHG has great potential applications in self-powered wireless sensor networks (WSNs) for environmental monitoring of intelligent agriculture.

## 1 Introduction

Wireless sensor networks (WSNs), comprising a multitude of processors, sensors, and radio nodes, promote the information and intelligent evolution of human society. This sophisticated network finds widespread application across diverse domains, including industrial control, smart home systems, consumer electronics, security equipment, logistics infrastructure, intelligent agriculture, environmental perception, and health monitoring systems. However, the efficacy of battery-powered sensors within WSNs faces challenges due to inherent limitations in commonly employed batteries, such as lithium-ion and fuel cells. These limitations encompass constraints related to energy storage capacity, portability, and environmental impact, thereby posing significant hurdles to endurance, operational efficiency, and reliability.^1−4^ Consequently, researchers are engaged in investigating methodologies to harness wind energy from the ambient environment with the goal of establishing self-powered WSNs. Traditional wind generators, characterized by their substantial weight and volume, remote installation, and elevated manufacturing and installation costs, have constrained their widespread adoption in self-powered WSNs. Miniaturized wind generators, which rely on the principle of electromagnetic induction, represent a prevalent alternative. Despite their merits, including a simple structure, a high electromechanical coupling coefficient, and simple processing, these generators exhibit suboptimal power generation efficiency under conditions of low wind speed.^5,6^

Triboelectric nanogenerator (TENG) invented by Wang’s group can adeptly convert mechanical energy into electrical energy through the synergistic mechanisms of contact electrification and electrostatic induction. Distinguished by its cost-effectiveness and broad material selectivity, the TENG exhibits notable advantages. Of particular significance is its high energy conversion efficiency, especially under low-frequency stimuli, which enables it being a promising and viable alternative for powering WSNs by harvesting breeze wind energy.^7^ Typical structures of TENG for wind energy harvesting can be divided into two categories based on their generation mechanisms. The first category is the rotation-sliding mode, such as rotation-disk,^8−11^ rotation-cylinder,^12,13^ and rotation-blade architectures.^14−16^ To mitigate the challenges associated with rigid contact, namely, friction and wear, scholars have proposed innovative designs featuring soft contact. These include the revision of dielectric material arrangements^17−19^ and the incorporation of unconventional tribomaterials such as rabbit hair,^20,21^ Ag fiber cloth,^22^ and cotton.^23^ The second one is vertical contact-separation (CS) mode.^24−26^ To augment generation efficiency under low wind speeds, scholars also explored novel structures tailored for breeze wind energy harvesting.^27−30^ Particularly, the integration of wind-induced vibration with TENG facilitates the contact and separation between tribomaterials and consequently lowers the start-up wind speed.^31−35^ It is evident that the rigid contact in the rotation-sliding mode gives rise to non-negligible frictional resistance and wear. In contrast, the challenge associated with soft contact lies in optimizing the effective contact area between tribomaterials, which determines the quantity of tribo-charge. The vertical CS mode ensures a substantial effective contact area, but the low operating frequency leads to large capacity reactance, high cost of matched load resistance, but poor generating performance. Consequently, there exists a compelling need to develop efficient TENGs for capturing breeze wind energy.

In this study, we present a novel blade-type triboelectric-electromagnetic hybrid generator (BT-TEHG) constructed from blade-type TENG units and a rotating disk electromagnetic generator (EMG). The novelty of our work lies in a double frequency up-conversion (DFUC) mechanism through the systematic arrangement of the TENG units and multiple plectrums. This configuration is conducive to heighten output power, particularly at low wind speeds. The collision between two tribomaterials amplifies the effective contact area, consequently elevating the charge density on the contact surfaces and mitigating frictional wear. To improve output power, we conducted parameter optimizations of the TENG and EMG. The generating properties of the BT-TEHG were experimentally measured. At wind speeds of 2.2 and 10 m/s, the BT-TEHG can output average power of 1.30 and 19.01 mW, respectively. Furthermore, it was used to charge capacitors, light up light emitting diodes (LEDs), and power commercial wireless sensors through an energy management module. These demonstrations validate its potential utility as a distributed power source for WSNs.

## 2 Results and Discussion

### 2.1 Structure Design and Working Principle

**Figure 1:**
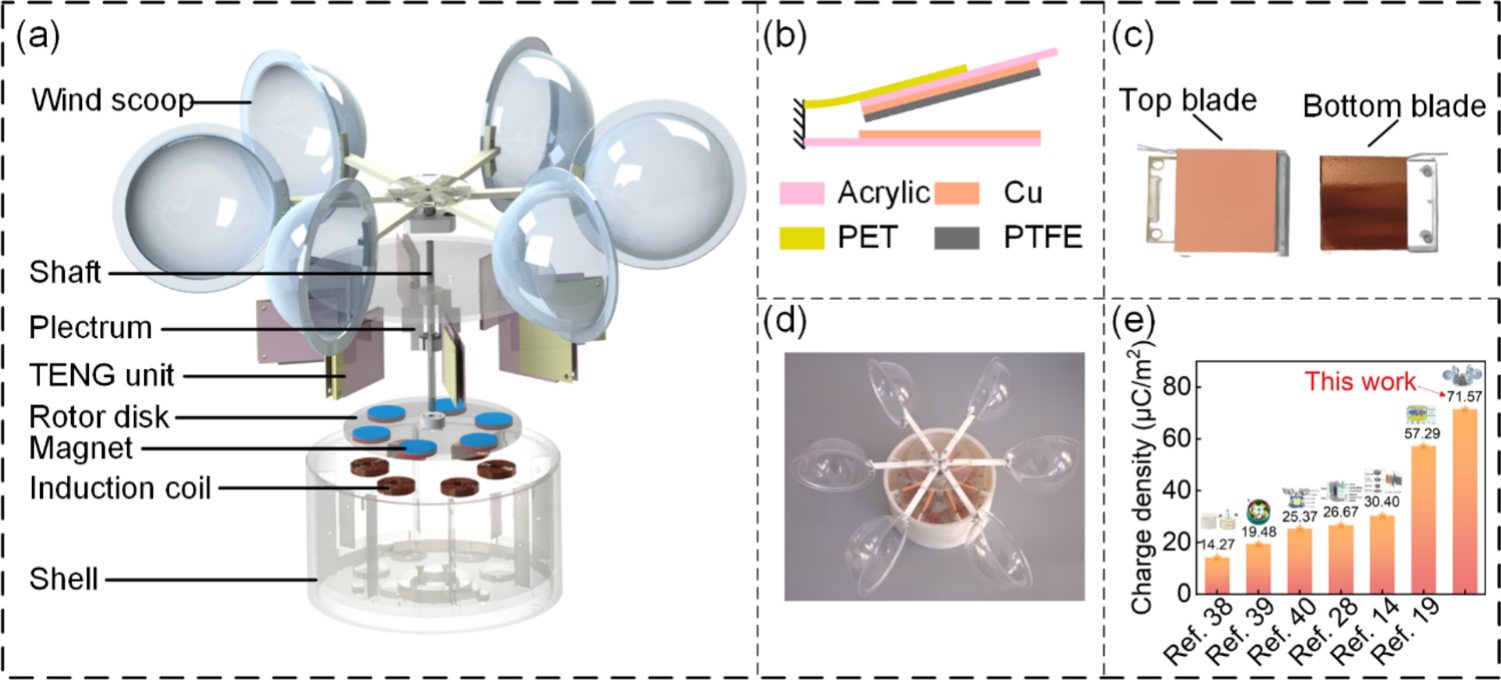
(a) Explosion diagram of the blade-type triboelectric-electromagnetic hybrid generator (BT-TEHG). (b) Schematic of the blade-type triboelectric nanogenerator (TENG) unit. (c) Photograph of two blades of a TENG unit. (d) Prototype of the BT-TEHG. (e) Comparison of surface charge density between BT-TEHG and other works.

The BT-TEHG with a DFUC mechanism is illustrated in Figure 1. It comprises eight blade-type TENG units and a rotating disk EMG enclosed within a shell (Figure 1a). This enclosure protects against environmental disturbances, such as humidity and dust, ensuring operational stability and compactness. Figure 1b provides a detailed schematic of the blade-type TENG unit, employing poly(tetrafluoroethylene) (PTFE) and copper as the tribomaterials. The bottom blade features a copper foil affixed to an acrylic substrate, acting as a stationary contact electrode. Correspondingly, a copper foil positioned between a PTFE film and an acrylic substrate is named as a rotatable back electrode, constituting the top blade with a poly(ethylene terephthalate) (PET) sheet together. Figure 1c showcases a photograph of two blades of a TENG unit. The prototype of the proposed BT-TEHG is presented in Figure 1d. In this device, the breeze wind energy is collected by an acrylic wind scoop and converted into rotational energy of shaft, which in turn drives the plectrums to rotate. The rotating plectrums pluck the top blades and then release them, resulting in periodic contact and separation between top and bottom blades, thus generating an alternative current.

Through the interplay between plectrums and TENG units, a double frequency up-conversion (DFUC) mechanism is introduced to achieve high-frequency responses under breeze wind excitation. In this context, four plectrums are employed to modulate the plucking frequency, achieving a quadrupled frequency relative to the rotating shaft, denoted as the first frequency up-conversion (FUC). Upon the release of the separated top blade, it collides with the bottom one at the natural frequency of the PET sheet, which surpasses the trigger frequency, termed the second FUC. This design yields numerous advantages: it facilitates an augmentation in the CS frequency between two triboelectric layers, thereby mitigating the capacitive reactance and matched resistance of the TENG, ultimately resulting in elevated output power.^36^ The CS mode concurrently attenuates the friction resistance and wear between triboelectric layers, thereby prolonging the operational lifespan of the system. Furthermore, the collision promotes an expansion of the effective contact area between two triboelectric layers, engendering a higher surface charge density and enhancing overall output performance.^37^ As shown in Figure 1e, since the TENG works on the principle of contact electrification and electrostatic induction, the surface charge density on the triboelectric layer is an important indicator to measure the output performance of the generator. Benefiting from the novel design, a TENG unit generates a higher charge density of 71.57 μC/m^2^ than other works in recent years.^14,19,28,38−40^ In addition, previous studies have demonstrated that the TENG generates higher energy conversion efficiency than EMG under breeze wind stimuli. In contrast, the EMG dominates power generation at high-speed wind condition.^41^ Accordingly, the BT-TEHG takes advantage of the complementary nature of TENG and EMG under different wind speeds, achieving synergistically efficient power generation within a wide range of wind speeds.

**Figure 2:**
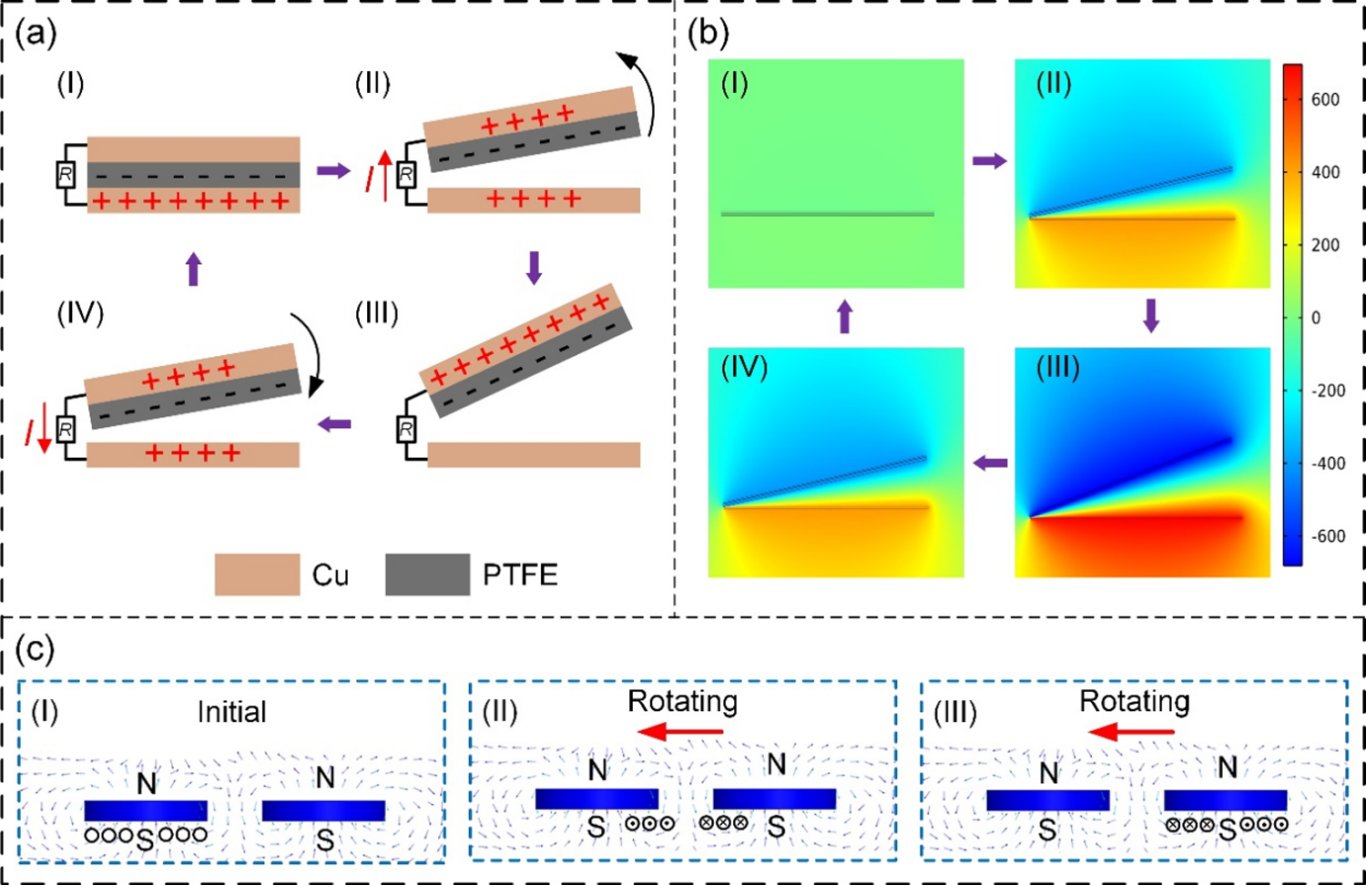
Working principle of the BT-TEHG: (a) Working principle of the TENG. (b) Surface potential distribution of a TENG unit during contact-separation operation. (c) Working principle of the electromagnetic generator (EMG).

The electricity generation process of the BT-TEHG can be divided into the TENG and EMG parts. Illustrated in Figure 2a, the operating principle of TENG is based on triboelectric and electrostatic induction effects. In the initial state (Figure 2a(I)), electrons undergo transfer from the copper foil of the contact electrode to the PTFE film due to contact electrification, instigating net negative charges at the PTFE film surface and an equivalent number of positive charges on the copper foil surface.^42^ Notably, an absence of an electric potential difference between back and contact electrodes characterizes this stage. Upon the plucking of the top blade by the plectrum, the PTFE film undergoes separation from the contact electrode (Figure 2a(II)). This separation prompts a potential difference between the two electrodes, forming an instantaneous current as electrons migrate from the back electrode to the contact electrode. At the maximum separation angle, all positive charges aggregate on the back electrode (Figure 2a(III)). Subsequently, as the plectrum continues its rotation, the top blade is released. During the reduction of the separation distance, electrons transition from the contact electrode back to the back electrode, producing a reverse instantaneous current (Figure 2a(IV)). Upon recontact between the PTFE film and the contact electrode, all induced charges are neutralized (Figure 2a(I)). Due to the continuous rotation of plectrums, a periodic alternating current is engendered from the TENG units in this cyclical process. To acquire a more intuitive comprehension of the potential distribution between two triboelectric layers, a finite element simulation is conducted utilizing COMSOL Multiphysics 6.0. The outcomes depicted in Figure 2b align with the stages in Figure 2a. Obviously, a discernible correlation exists between the separation angle and the electric potential difference.

Figure 2c describes the operational principle of the EMG based on electromagnetic induction. In the initial state (Figure 2c(I)), the magnet within the rotor disk is aligned with the induction coil, maintaining a constant magnetic flux within the coil, which outputs no current. Upon the rotation of the rotor disk, the magnet’s position relative to the induction coil varies, inducing a flux change. Consequently, a current is generated in the coil, according to Lenz’s law (Figure 2c(II)). As the rotor disk persists in its rotation, the magnetic flux traverses the coil from the opposing direction, leading to an induced current in the reverse direction (Figure 2c(III)). This cyclic process realizes a complete cycle of alternating current within the EMG.

### 2.2 Modeling

**Figure 3:**
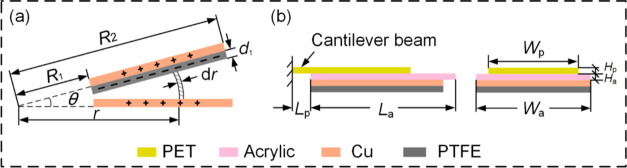
(a) Nonparallel plate capacitor model for TENG. (b) Dimensional parameters of the top blade.

In order to describe theoretically the electrical characteristics of the TENG part, a nonparallel plate capacitor model filled with air and PTFE film is established based on the specific configuration, as depicted in Figure 3a. The thickness and dielectric constant of PTFE are *d*_1_ and *ε*_1_, respectively. The separation angle, amount of transferred charge, and potential difference between two electrodes are defined as *θ*, *Q*, and *V*, respectively. The surface tribo-charge density of the PTFE film is *σ*. Since the contact area (*S*) between two blades is much larger than their separation distance in the experiments, an approximate analytical *V*–*Q*–*θ* relationship can be derived by neglecting the edge effect. Under ideal conditions, the nonparallel plate capacitor can be divided into multiple parallel plate microcapacitors connected in parallel. Known that the capacitance of a parallel plate capacitor with air as dielectric is *C* = *ε*_0_*S*/*d*, where *d* is the separation distance between two plates. Accordingly, the capacitance of the blade-type TENG can be calculated as
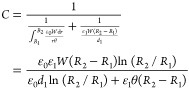
1where *W* and (*R*_2_ – *R*_1_) are the width and length of the contact area.

According to Gauss’s theorem, the electric field strength of a microcapacitor in each medium at the position of *r* is expressed as follows
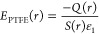
2
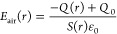
3where *Q*_0_ is the amount of triboelectric charge. The voltage between the two electrodes is *V*(*r*) = *E*_PTFE_*d*_1_ + *E*_air_*rθ*. Since there is no charge transfer (*Q* = 0) in the open-circuit (OC) state, the OC voltage is given by
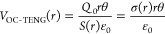
4Since the total tribo-charge is unchanged, which is expressed as
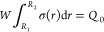
5Substituting eq 4 into eq 5, the OC voltage of TENG is obtained as
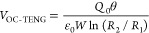
6According to the electrical potential superposition principle, the *V*–*Q*–*θ* relationship can be given by
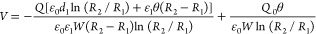
7Under short-circuit (SC) condition, the transferred charges and SC current can be derived as
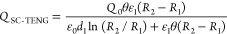
8
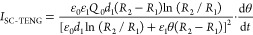
9Since the TENG can be simplified to be a serial connection of an ideal voltage source and a capacitor, its average impedance is approximately *X*_g_ = 1/(*ωC*_avg_), where *ω* is the angular frequency of the signal source and *C*_avg_ is the average inherent capacitance. When it is externally connected with a load resistance *R*_L_, the power delivered to the load is calculated as^43^
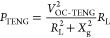
10Therefore, the optimal power can be derived by letting
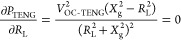
11Obviously, the matched resistance for the optimal power depends on the average impedance *X*_g_. Furthermore, all approaches that can lower the average impedance *X*_g_ are conducive to reducing the matched resistance and increasing the output power of the TENG, including expanding contact area, amplifying response frequency, and parallel connection of multiple TENGs.^36^ These equations theoretically demonstrate the advantages and benefits of the proposed BT-TEHG.

The detailed schematic diagram of the top blade is shown in Figure 3b. Herein, the PET sheet acts as a cantilever beam with length (*L*_p_), width (*W*_p_), and height (*H*_p_), while the acrylic substrate possesses dimensions of length (*L*_a_), width (*W*_a_), and height (*H*_a_). As mentioned above, the top blade will impact the bottom one during the DFUC mechanism, which in turn generates a contact force. It is worth noting that the contact force introduces surface deformation and changes the microscopic contact area and interfacial dangling bonds of the tribomaterials, ultimately improving the surface charge density and the amount of transferred charge. Therefore, the contact force is a key factor to affect the triboelectric behavior and electrical output, whose maximum is described as^44^
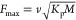
12where *v* and *M* are impact velocity and equivalent mass of the top blade, respectively, and *K*_p_ is the equivalent stiffness of the cantilever beam, which can be expressed as^45^
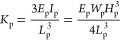
13where *E*_p_ and *I*_p_ are the Young’s modulus and moment of inertia of the PET sheet, respectively. It predicts that the maximum contact force is proportional to *v*, *K*_p_, and *M*.

To analyze the dynamic responses of the top blade, it is simplified to be a lumped-parameter model, as illustrated in Figure S1. At the initial stage (Figure S1a), the top blade is plucked away from the static position and moves along with the plectrum until their overlap length is reduced to zero, in which case the plectrum rotates at a constant angular velocity *ω*. Within the contact time *t*, the moving speed is *ż* = *L*_1_*ω* cos(*ωt*), 0 ≤ *t* ≤ *t*_f_, where *L*_1_ is the rotational radius of plectrum and *t*_f_ is the separation time. When they get separated, the blade undergoes free vibration (Figure S1b) and the governing equation can be expressed as
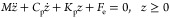
14where *F*_e_, *C*_p_, and *z* are the electrostatic force, mechanical damping coefficient, and the relative displacement of the top blade tip relative to the initial static position, respectively.

When the top blade collides with the bottom one or plectrum, its moving speed changes at the moment of impact, which can be described by *ż*^+^ = −*e*_*i*_*ż*^–^ (*i* = 1, 2), where *e*_1_ and *e*_2_ are the coefficients of restitution between two blades and between the top blade and plectrum, respectively, and *ż*^+^ and *ż*^–^ denote the moving speeds of the top blade just after and before impact, respectively. At the moment that the top blade is just in contact with plectrum, a contact force *F* = *Mz̈* + *C*_p_*ż* + *K*_p_*z* + *F*_e_ (*z* > 0) is exerted on the blade. When the value is zero, it indicates the state of separation.

With respect to the EMG part, the OC voltage (*V*_OC-EMG_) and SC current (*I*_SC-EMG_) can be expressed as
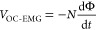
15
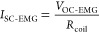
16where *N* is the number of turns of the induction coil, Φ is the magnetic flux, and *R*_coil_ is the internal resistance of the coil. Hence, the performance of EMG is determined by the rate of change of magnetic flux, *N*, and *R*_coil_.

### 2.3 Performance

**Figure 4:**
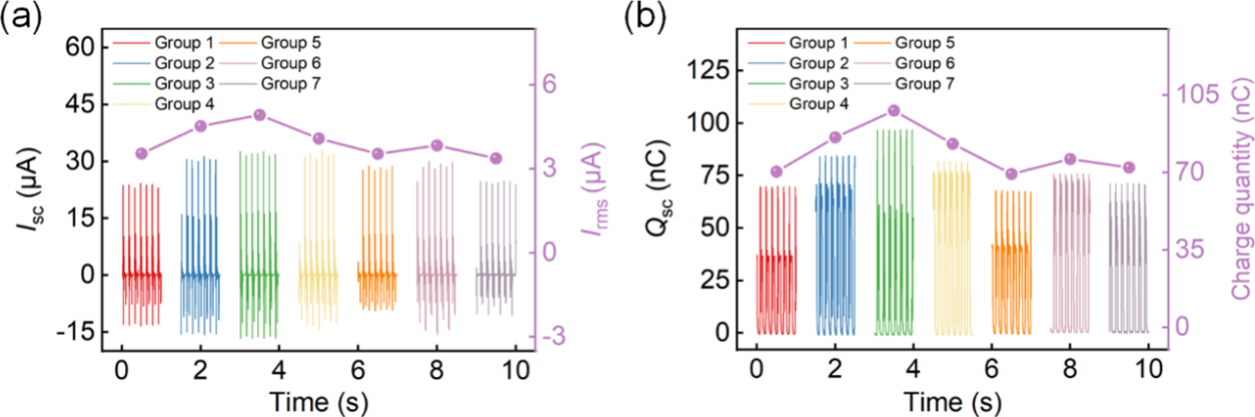
Output performance of 7 groups of TENGs with different parameters at a rotational speed of 100 rpm. (a) Short-circuit (SC) current *I*_SC_ and its root-mean-square (RMS) value *I*_rms_. (b) SC transferred charge *Q*_SC_ and transferred charge quantity.

To investigate the influences of dimensions on output characteristics and determine optimal parameters of the top blade, 7 groups of TENGs consisting of different sizes of the PET sheet and acrylic substrate are produced. The detailed dimension parameters are given in Table S1. It should be noted that only the length (*L*_p_) and width (*W*_p_) of the cantilever beam and the height (*H*_a_) of the acrylic substrate are variable, so as to simplify the optimization procedure. The shaft of the BT-TEHG is connected to a DC motor, offering tunable rotational speeds. The motor test platform is depicted in Figure S2. Under a constant rotational speed of 100 rpm, the output SC current (*I*_SC_) and transferred charge (*Q*_SC_) were measured and are compared in Figure 4. For Groups 1, 2, and 3 with constant *W*_p_ and *H*_a_, the root-mean-square (RMS) current (*I*_rms_) and transferred charge quantity exhibit a gradual increase with the augmentation of *L*_p_. This behavior is attributed to the elongation of the cantilever beam, leading to an expanded overlap length for the plectrum and acrylic substrate, thereby increasing the separation distance between the top and bottom blades. Consequently, more elastic potential energy is accumulated in the cantilever beam and ultimately converted into more electrical energy. Nonetheless, an overextended cantilever beam will pose an obstacle to the rotation of plectrum, culminating in the failure of the DFUC mechanism. When maintaining constant values for *L*_p_ and *W*_p_, an augmented height *H*_a_ of the acrylic substrate results in amplified *M* and enhanced contact force between the two blades, as described in eq 12. This significantly expands the effective contact area between two triboelectric layers, introducing an ascending surface charge density and improved generating performance, as evidenced in the outcomes of Groups 3, 4, and 5. Although there is no *W*_p_ in the expressions of *I*_SC_ and *Q*_SC_, the comparative analysis among Groups 3, 6, and 7 reveals that a broader cantilever beam introduces greater stiffness and more elastic potential energy at the same separation distance, consequently achieving more electrical output. Experimental findings demonstrate that at dimensions *L*_p_ = 5 mm, *W*_p_ = 50 mm, and *H*_a_ = 3 mm, the maximum *I*_rms_ attains 4.93 μA, accompanied by a transferred charge quantity of 98.02 nC. Hence, the size parameters of Group 3 are designated as the benchmark for TENG.

**Figure 5:**
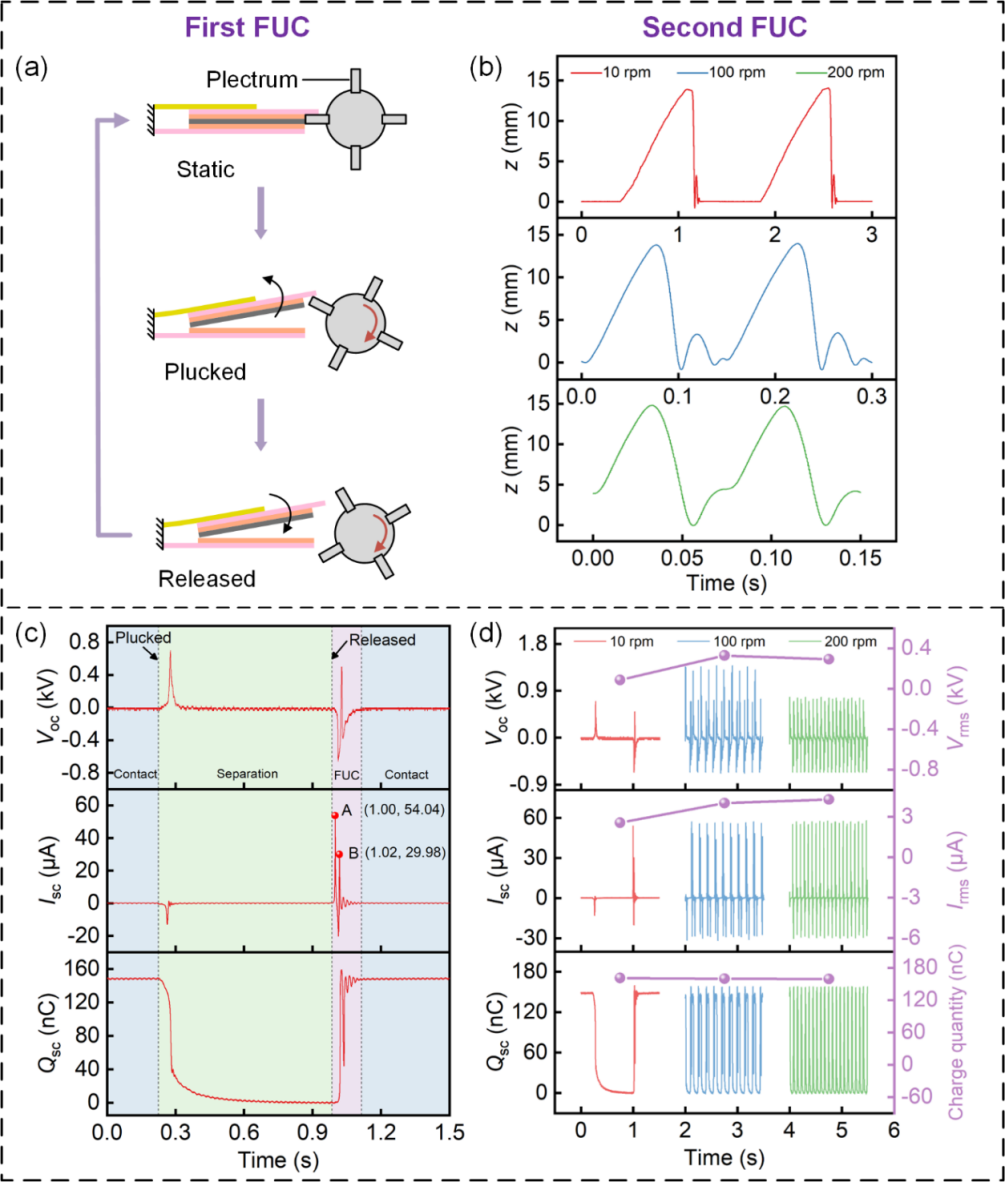
(a) Interaction between the rotational plectrum and the top blade realizes the first FUC. (b) Displacement responses of the top blade when plucked twice at different rotational speeds. (c) Electrical characteristics of a TENG unit at a rotational speed of 10 rpm. (d) Comparison of electrical characteristics at different rotational speeds.

In order to assess the impact of the DFUC mechanism on the dynamic response and output performance, the electrical characteristics of a TENG unit are measured and compared at different rotational speeds. Figure 5a presents the interaction between the rotational plectrum and top blade, realizing four pluckings within one cycle of rotating shaft. This process is named as the first FUC. Prior to the release of the top blade, elastic potential energy is stored in the cantilever beam, which is then converted into kinetic energy upon release. Figure 5b depicts the displacement responses (*z*) of the top blade when it is plucked twice at different rotational speeds. For the speeds of 10 and 100 rpm, two distinct CS processes can be observed after each plucking, revealing two primary inelastic collisions. Despite part of kinetic energy dissipated in the forms of acoustic, thermal, potential energy, etc. during the first collision, the residual is still sufficient to overcome the effect of electrostatic adsorption force and cause the top blade to rebound. More importantly, the vibration frequency after being released is much higher than the plucking frequency, achieving high-frequency response under low-frequency excitation, denoted as the second FUC. However, when the speed reaches 200 rpm, the speed is so fast that there is no time for a secondary collision. Correspondingly, the OC voltage (*V*_OC_), SC current (*I*_SC_), and transferred charge (*Q*_SC_) at a rotational speed of 10 rpm are shown in Figure 5c. During the contact state, no charge transfer occurs under SC conditions, and there is an absence of potential difference between the two electrodes. Once the plucking happens, the separation of two triboelectric layers ensues, promoting a substantial charge transfer and forming an instantaneous current. This process exhibits a rapid saturation trend. Notably, the output characteristics manifest a high sensitivity to the initial separation distance. The transferred charge quantity is 161.03 nC, yielding a charge density of 71.57 μC/m^2^. The peak–peak values of *V*_OC_ and *I*_SC_ are 1.36 kV and 69.00 μA, respectively. Although the trend of the *V*_OC_ waveform resembles that of *I*_SC_, it deviates from the trend outlined in eq 6. This difference may be caused by the smaller input impedance of the oscilloscope than the internal impedance of TENG, resulting in charge flowing through the instrument. Therefore, the measured *V*_OC_ represents the voltage across divider resistor in the oscilloscope.^46^ After the top blade is released, the FUC phenomenon also appears in the electrical signal. Taking the waveform of *I*_SC_ as an example, the time interval between two peaks A and B is 0.02 s. That is to say, the vibration frequency of the top blade is approximately 50 Hz, a frequency 300 times greater than the rotational frequency of the shaft (0.17 Hz). As a result, the heightened CS frequency diminishes the impedance of TENG, thereby amplifying the output power, which is a notable advantage conferred by the DFUC mechanism.^36^ At last, the top blade is again in contact with the contact electrode as the kinetic energy disappears.

Figure 5d provides the electrical characteristics at varying rotational speeds. Obviously, the RMS value *I*_rms_ exhibits a gradual increment alongside a concurrent reduction in the transferred charge quantity with escalating rotational speed. In contrast, the RMS voltage *V*_rms_ experiences an initial increase, followed by a subsequent decline. Such divergent trends can be attributed to the following reasons. As shown in Figure 5c, it takes about 0.761 s for charge transfer to reach the saturation state. With the increase in the rotational speed, the time devoted to charge transfer within each plucking period gradually decreases, resulting in a gradual reduction in the transferred charge quantity. However, *I*_rms_ represents the amount of transferred charge in unit time. Since the induced charge is highly sensitive to the initial separation distance, the transfer time for 80% charges is only 0.077 s, close to the plucking cycle (0.075 s) at 200 rpm. Therefore, *I*_rms_ shows an upward trend with the enhancement of the plucking frequency or rotational speed. As expressed in eq 7, the voltage is related to the amounts of tribo-charge *Q*_0_ and transferred charge *Q*. Within the rotational speed range of 10 to 100 rpm, charge transfer plays the predominant role. However, at the speed of 200 rpm, there is only one impact caused by one plucking and its contribution to triboelectricity is weakened. The inadequate contact between two triboelectric layers leads to a pronounced reduction in tribo-charge and an ultimately declining trend in *V*_rms_. Most importantly, at this rotational speed, the vibrational frequency of the top blade persists at approximately 15.9 times the rotational frequency of the shaft, thereby affirming the validity and feasibility of the DFUC mechanism.

**Figure 6:**
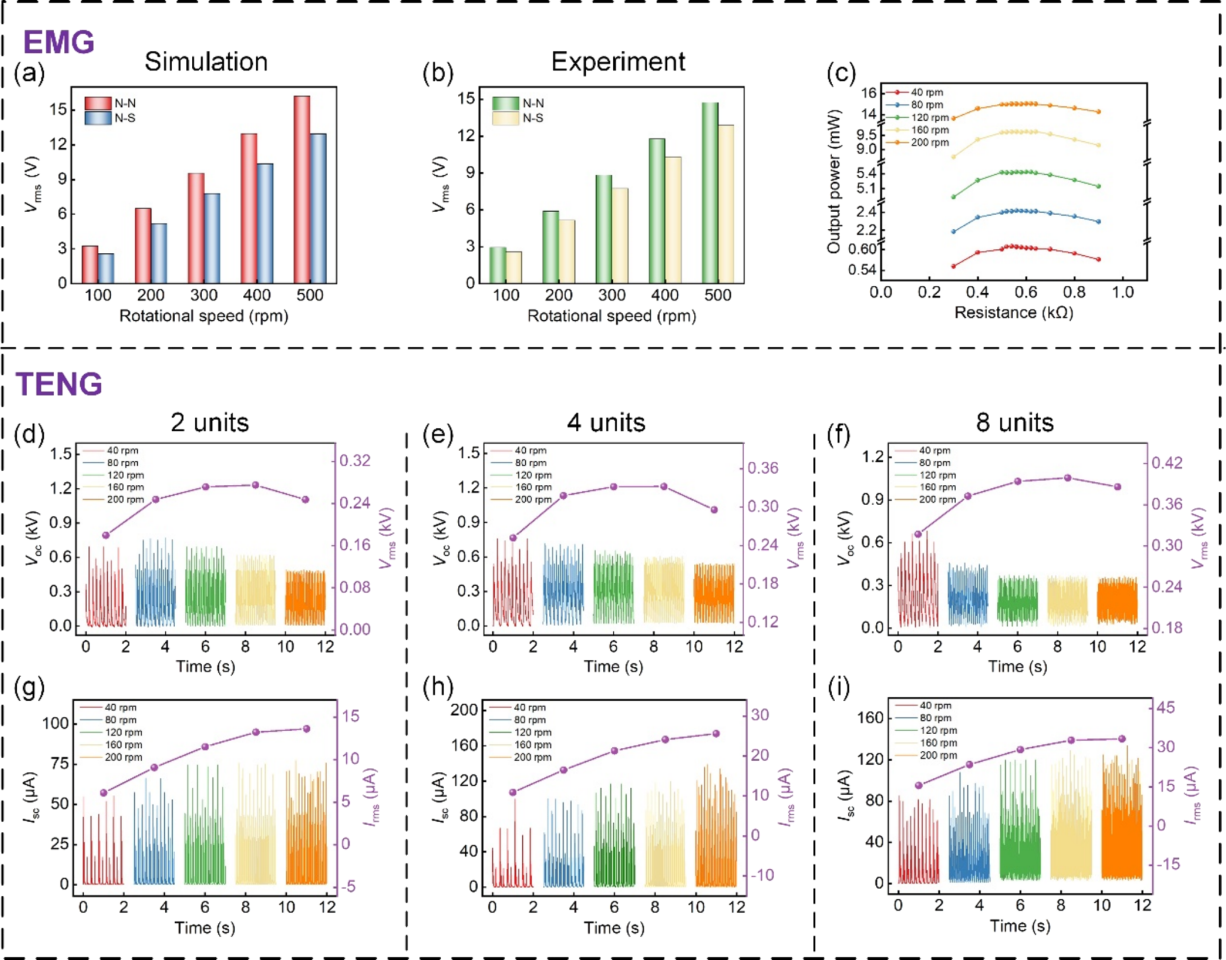
(a) Simulated *V*_rms_ from the EMG at different rotational speeds. (b) Measured *V*_rms_ from the EMG at different rotational speeds. (c) Output power of EMG with different load resistances. (d–f) *V*_OC_ and *V*_rms_ from different TENG units in parallel connection at different rotational speeds. (g–i) *I*_SC_ and *I*_rms_ from different TENG units in parallel connection at different rotational speeds.

To assess the effect of magnetization direction of adjacent magnets on the output performance of the EMG, we investigated the *V*_rms_ across the induction coil under two conditions: the same magnetization direction (N–N) and different magnetization directions (N–S) (Figure S3). The power generation characteristics at varying rotational speeds were simulated and measured, as presented in Figure 6a,b. The investigation disclosed a progressive increase in *V*_rms_ with the rotational speed, regardless of the magnetization direction. However, the values under the same magnetization direction consistently surpassed that under different magnetization directions. Consequently, for subsequent experiments, magnets were arranged in the same magnetization direction. Furthermore, the output power across load resistances connected to the induction coil was measured at different rotational speeds, as illustrated in Figure 6c. The findings indicated a gradual rise in matched load resistance from 540 to 620 Ω as the rotational speed ranged from 40 to 200 rpm. This value was comparable to the internal resistance of the coils in series (578 Ω). The observed change was attributed to the variation of the inductive reactance, which is proportionate to the rotational speed.

Figure 6d–i present the rectified *V*_OC_ and *I*_SC_ for 2, 4, and 8 TENG units in parallel connection at different rotational speeds. All units are distributed evenly around the circumference. Consistent trends are observed in both the voltage and current. As the rotational speed increases from 40 to 200 rpm, *I*_rms_ rises monotonically and levels off at 200 rpm. In contrast, *V*_rms_ initially ascends and subsequently descends. This phenomenon may be attributed to the escalating CS frequency with an increase in rotational speed, resulting in a general rise in *I*_rms_. However, when the plucking frequency of the plectrum is high enough, there is insufficient time for adequate contact. This leads to a reduction in the effective contact area and tribo-charges, causing a downward trend in *V*_rms_ and saturation in *I*_rms_. It also reveals that more units in parallel connection correspond to increased *V*_rms_ and *I*_rms_ at the same rotational speed, indicating enhanced generating performance. Nonetheless, it is imperative to consider the limitations of the shell’s capacity and the operational space of the TENG. Within these constraints, this design can accommodate up to 8 TENG units.

**Figure 7:**
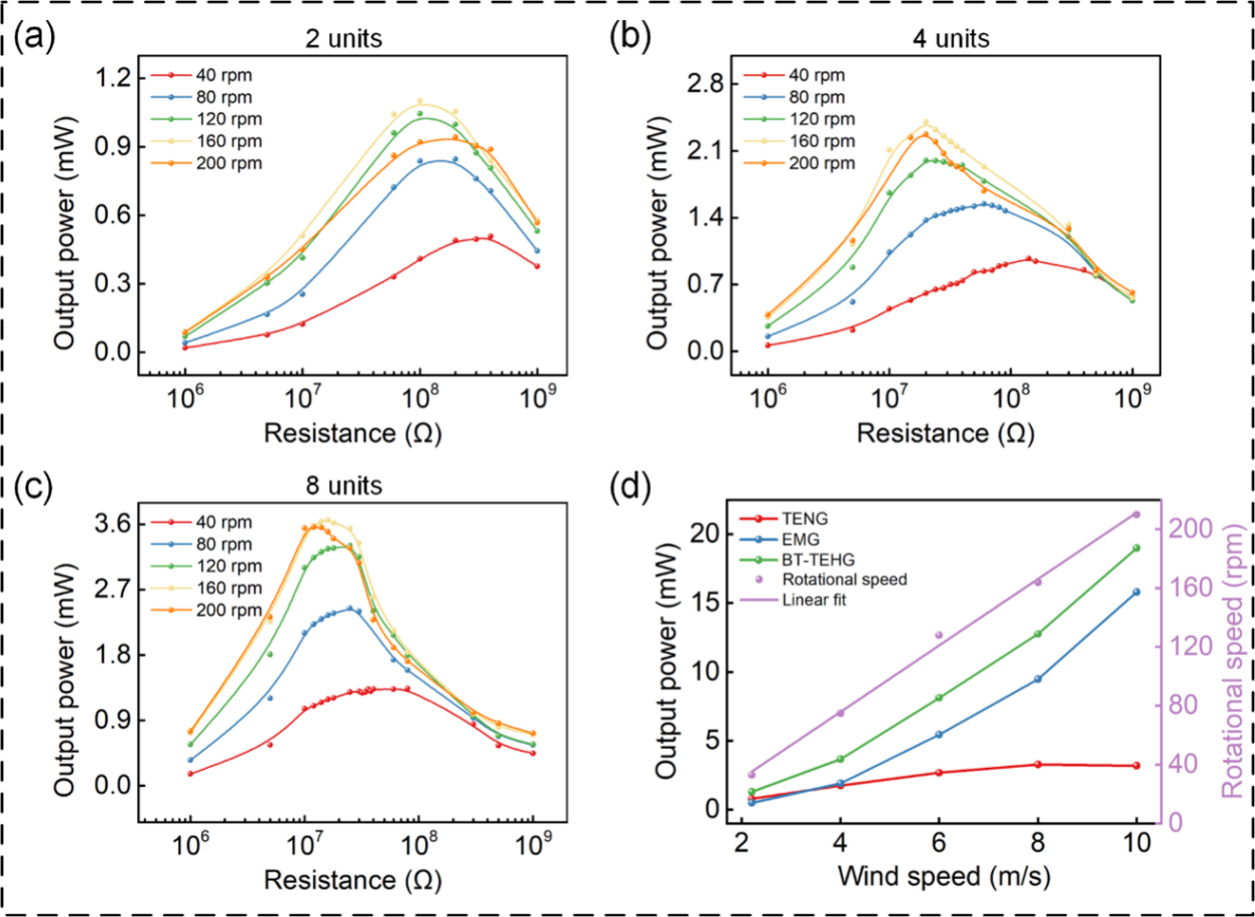
(a–c) Output power of 2, 4, and 8 TENG units with different load resistances at distinct rotational speeds. (d) Output power of the BT-TEHG, TENG part, and EMG part and the corresponding rotational speeds at different wind speeds.

The output characteristics of the standalone TENG and the BT-TEHG are critical to evaluate their generating performance. First, we investigated the output power from 2, 4, and 8 TENG units connected to varying load resistances under diverse rotational speeds (Figure 7a–c). Irrespective of the specific quantity of TENG units, the output power exhibits a rising trend followed by a decline with increasing load resistance, reaching its maximum when connected to the matched resistance. Concurrently, as the rotational speed increases, the maximum output power initially ascends and subsequently descends, but the corresponding matched resistance gradually decreases. The fluctuations observed in output power are caused by changes in tribo-charges and transferred charge quantities at distinct rotational speeds. The reduction of matched resistance is induced by the decrease of capacitive reactance that is inversely proportional to the rotational speed, as described in eq 11. The maximum output powers yielded by 2, 4, and 8 TENG units amount to 1.10, 2.40, and 3.66 mW, respectively. The associated matched resistances stand at 100, 20, and 16 MΩ. Throughout the experiments, the optimal rotational speed consistently remained at 160 rpm. By comparing the matched resistances of the three groups, it can be found that the greater the number of TENG units connected in parallel, the smaller the matched resistance. This is because the TENG can be considered as a capacitor, where parallel connections serve to mitigate capacitive reactance, subsequently leading to a decrease in matched resistance. In short, augmenting the rotational speed and implementing parallel connections are effective strategies for reducing matched resistance. When connected to the matched resistances, the output power of 2, 4, and 8 standalone TENG units is compared with that of the standalone EMG, as shown in Figure S4.

Figure 7d shows the output power of the BT-TEHG connected to the matched resistances and the corresponding rotational speed in the actual wind field. The corresponding output power from TENG and EMG parts are also presented. Obviously, output powers steadily increase with the increase of wind speed but with different slopes. As the wind speed rises, the contribution of TENG to the total output power gradually decreases, and EMG gradually takes the upper hand. At a wind speed of 2.2 m/s (about 33 rpm), the BT-TEHG can steadily generate power of 1.30 mW and the value reaches to 19.01 mW at 10 m/s (about 210 rpm), indicating better output performance compared to standalone TENG and EMG. Correspondingly, the maximum energy conversion efficiency of TENG part is 23.01% at a wind speed of 2.2 m/s, while that of the EMG part is 3.80% at 10 m/s. The detailed values are listed in Table S2, and the calculation is shown in Note S1. The results indicate that the efficiency of the plucked TENG is superior to that of previous TENGs using solid–solid CS mode,^47−50^ demonstrating the advantage of DFUC mechanism in improving energy conversion efficiency. The relationship between wind speed and rotational speed is linearly fitted as *Y* = 22.25*X* – 14.45, where *X* is the wind speed and *Y* is the rotational speed. Accordingly, the wind speed corresponding to the optimal rotational speed of the TENG (160 rpm) is about 7.84 m/s. Moreover, the relationship between output power and rotational speed is close to the data measured in the motor test platform, demonstrating the robustness of the generating performance of the BT-TEHG.

## 3 Demonstration

**Figure 8:**
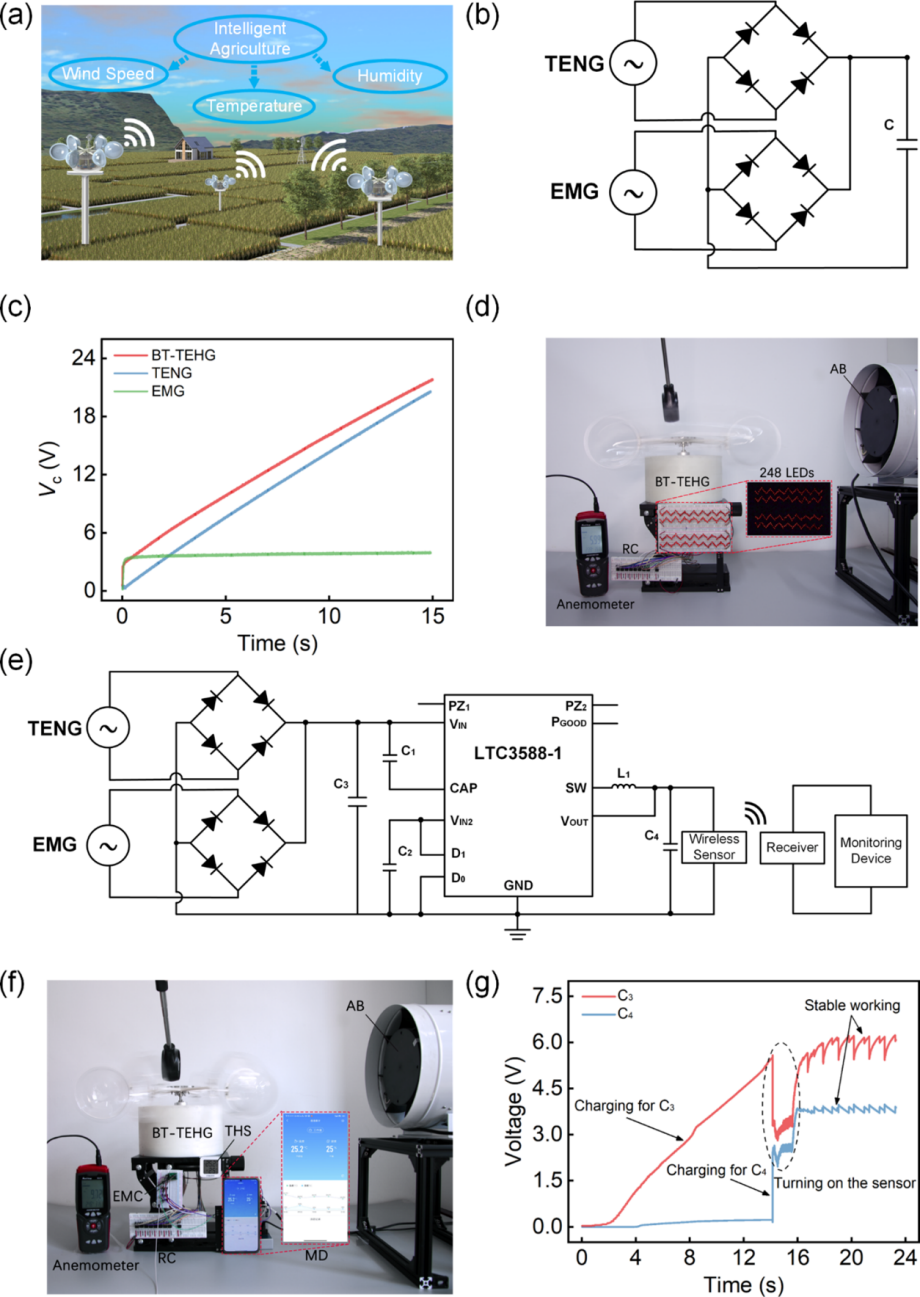
(a) Application of the BT-TEHG in intelligent agriculture. (b) Rectification circuit for charging capacitor. (c) Capacitor charging curves of the TENG, EMG, and BT-TEHG. (d) Photo of 248 LEDs in series powered by the BT-TEHG. (e) Circuit connection diagram of a self-powered wireless monitoring system. (f) Wireless temperature and humidity monitoring system powered by the BT-TEHG. (g) Charging cures of C_3_ and C_4_.

Figure 8a showcases the prospective application of BT-TEHG in the domain of intelligent agriculture. It collects and converts wind energy in the environment into electrical energy, thus providing distributed power supply for wireless sensor nodes in farmland areas and constructing self-powered WSNs. To demonstrate the capability to harvest wind energy and power electronic devices, the BT-TEHG consisting of eight TENG units is placed in the wind field generated by an air blower (AB) and the wind speed can be regulated by a controller. First, the charging capability of TENG, EMG, and BT-TEHG on a 22 μF capacitor is investigated at a wind speed of 6 m/s. A rectification circuit (RC) is employed to convert AC signals into DC outputs (Figure 8b). As shown in Figure 8c, EMG exhibits a faster charging rate than TENG before approaching the saturation voltage of 4 V. The charging curve of the TENG is almost linear, and the voltage exceeds that of the EMG in 2.3 s. The BT-TEHG shows the best charging capacity and possesses the characteristics of the first two. The voltage curve rises sharply in the initial stage and steadily thereafter, indicating the superiority of the hybrid energy harvesting mechanism. As presented in Figure 8d and Video S1, the BT-TEHG can light up 248 LEDs in series and 496 LEDs in parallel at a wind speed of 6 m/s, using the rectification circuit in Figure 8b. More importantly, the BT-TEHG assumes the role of a distributed power source for wireless sensors. Herein, a self-powered wireless temperature and humidity monitoring system is developed by connecting the BT-TEHG with a commercial temperature and humidity sensor (THS) through an energy management circuit (EMC). The detailed circuit connection diagram is displayed in Figure 8e. Within this system, the TENG and EMG are separately connected to rectification circuits, and the electrical energy is stored in input capacitor C_3_ (47 μF). Through the commercial LTC-3588 chip, a stable 3.3 V DC output voltage is stored in output capacitor C_4_ (47 μF), employed to energize the sensor equipped with a wireless communication module. Finally, the dynamic temperature and humidity fluctuations are monitored by a monitoring device (MD). Figure 8f and Video S2 present the operation of the self-powered monitoring system. The test results demonstrate the BT-TEHG’s pronounced potential as a distributed energy source for environmental monitoring of intelligent agriculture. The charging curves of C_3_ and C_4_ are shown in Figure 8g. In the initial stage, only C_3_ is charged, and the chip does not work. Until 5.5 V, the chip starts to rapidly charge C_4_, accompanied by a sharp decrease of voltage across C_3_. Once the voltage of C_4_ reaches the required working voltage of the THS, the sensor is turning on, and the communication with mobile phone is established through Bluetooth module. After charging at a wind speed of 10 m/s for 16 s, the voltages across C_3_ and C_4_ become quasi-periodic sawtooth pattern and the voltage of C_4_ locates around 3.8 V that is sufficient to support the sensor. As a result, the system can wirelessly monitor the temperature and humidity in the environment.

## 4 Conclusions

In this study, a novel blade-type triboelectric-electromagnetic hybrid generator (BT-TEHG) has been proposed for effectively capturing the breeze wind energy. A double frequency up-conversion (DFUC) mechanism was constructed to realize a high-frequency output from the TENG under low-frequency breeze wind excitation and ultimately augment the output power. Simulations and experiments were conducted to optimize the magnetization direction in the EMG and the dimensional parameters of the TENG unit. The generating characteristics of the BT-TEHG under varying rotational speeds and electrical boundary conditions were explored. Results show that the response frequency of the TENG can be amplified by 15.9 to 300 times when subjected to an excitation of rotational speed varying from 10 to 200 rpm. At wind speeds of 2.2 and 10 m/s, the BT-TEHG can generate, respectively, power of 1.30 and 19.01 mW when TENG and EMG are connected to the matched resistances. The optimal rotational speed for the TENG is 160 rpm, corresponding to a wind speed of 7.84 m/s. It also demonstrates the capabilities to quickly charge capacitors and feed 248 LEDs in series and 496 LEDs in parallel at a wind speed of approximately 6 m/s, indicating the superiority of the hybrid energy harvesting mechanism. Through an energy management circuit, a self-powered wireless temperature and humidity monitoring system was developed and powered by the BT-TEHG. In summary, this work demonstrates the potential application of the BT-TEHG as a distributed energy source for self-powered WSNs and provides promising application prospects in intelligent agriculture.

## 5 Experimental Section

### 5.1 Fabrication of the BT-TEHG

The shell with external dimensions of 200 mm (diameter) × 112 mm (height) is made from photosensitive resin using a three-dimensional (3D) printer. The materials of the wind scoops and shaft are acrylic and stainless steel, respectively. The diameter of the wind scoop is 120 mm, and the length of its moment arm is 120 mm. As for the top blade of the TENG, the PET sheet acts as the cantilever beam with dimensions of 5 mm × 50 mm × 0.3 mm. The acrylic substrate has dimensions of 55 mm × 58 mm × 3 mm. The PTFE film has dimensions of 49 mm × 58 mm × 0.3 mm. The copper foil has the dimensions of 49 mm × 58 mm × 0.1 mm. As for the bottom blade of the TENG, the copper foil has dimensions of 45 mm × 50 mm × 0.1 mm. As for the EMG, the dimensions of 6 induction coils are as follows: 0.2 mm wire diameter, 7.5 mm inner diameter, 29 mm outer diameter, 5 mm height, and 1600 turns. The gap between the magnet and coil surfaces is 2 mm. The material of the magnet is NdFeB (N35), and it has a diameter of 30 mm, height of 5 mm, and rotation radius of 50 mm. The acrylic rotor disk (diameter: 140 mm, thickness: 5 mm) is fabricated using laser cutting and contains six circular holes to accommodate magnets. The four acrylic plectrums have a rotation radius of 22 mm.

### 5.2 Electrical Measurement

The wind is produced by an air blower (SE-A250S, SENSDAR, China), and the speed is measured using a digital anemometer (RA410, SanLiang, China). The rotational speed is measured using a noncontact tachometer (UT373, UNI-T, China). The stable rotational speed is supplied by a DC motor (QW80BL00730450, CLKMOTOR, China). The voltage of EMG, current, and transferred charge of the TENG are measured using a programmable electrometer (6514, Keithley), and the data are recorded using a data acquisition system (NI 9229, National Instruments). LabVIEW is used to process and display the data. The voltage of the TENG is measured using an oscilloscope (MDO-2204EC, GWINSTEK, China). The potential distribution of the TENG is simulated using COMSOL Multiphysics 6.0 software. The torque of rotational shaft, tip displacement of the top blade, and elastic restoring force of cantilever beam are measured by using a dynamic torque sensor (DYN-200, DAYSENSOR, China), laser displacement sensor (HG-C1100, Panasonic, Japan), and dual-range force sensor (DFS-BTA, Vernier), respectively.

## Supporting Information Available

Lumped-parameter model of TENG involving mechanical and electrical parameters; detailed dimension parameters of top blade for different groups; schematic diagram of the motor test platform; arrangement of magnets; output power of the standalone TENG and standalone EMG under different rotational speeds when connected to the matched resistances; related parameters for energy conversion efficiency; calculation of energy conversion efficiency, and schematic diagram of the end displacement measurement of the cantilever beam (PDF)
BT-TEHG lighting up the LEDs (Movie S1) (MP4)
Wireless monitoring system powered by the BT-TEHG (Movie S2) (MP4)
