# Supplementary material for: A Blade-Type Triboelectric-Electromagnetic Hybrid Generator with Double Frequency Up-Conversion Mechanism for Harvesting Breeze Wind Energy
Source: ACS Appl Mater Interfaces. 2024 Jun 21;16(26):33404–15. doi: 10.1021/acsami.4c04377 (PMC11231971; doi:10.1021/acsami.4c04377)
Supplement: Supplementary file 1 — am4c04377_si_001.pdf [file am4c04377_si_001.pdf]

Supporting Information

A blade-type triboelectric-electromagnetic hybrid generator with double frequency up-conversion mechanism for harvesting breeze wind energy

Na Yang [a, ‡], Yingxuan Li [a, ‡], Zhenlong Xu [a, *], Yongkang Zhu [a], Qingkai He [a], Ziyi Wang [a], Xueting Zhang [a], Jingbiao Liu [a], Chaoran Liu [b], Yun Wang [a], Maoying Zhou [a], Tinghai Cheng [c, *], Zhong Lin Wang [c, *]

[a] School of Mechanical Engineering, Hangzhou Dianzi University, Hangzhou 310018, China
[b] Ministry of Education Engineering Research Center of Smart Microsensors and Microsystems, College of Electronics and Information, Hangzhou Dianzi University, Hangzhou 310018, China
[c] Beijing Institute of Nanoenergy and Nanosystems, Chinese Academy of Sciences, Beijing 101400, China

[‡] Na Yang and Yingxuan Li contributed equally to this work.

[*] Corresponding authors.

Email address: xzl@hdu.edu.cn; chengtinghai@binn.cas.cn; zlwang@binn.cas.cn

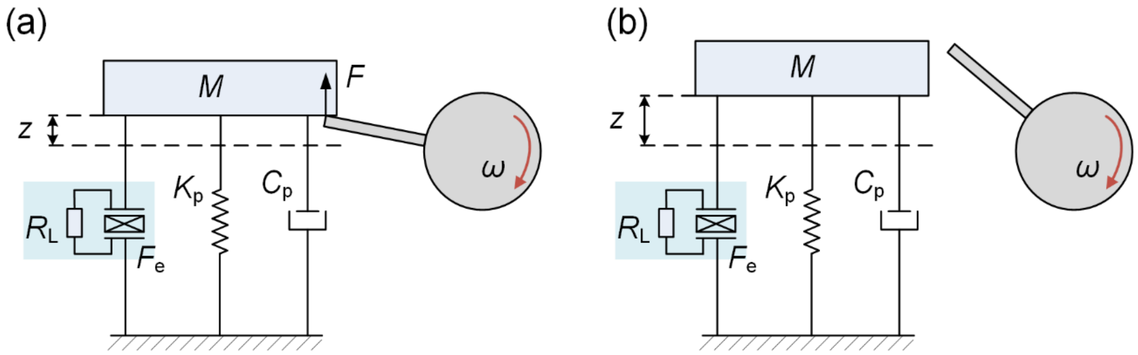

Figure S1. Lumped-parameter model of the TENG involving mechanical and electrical parameters. (a) Plucked status. (b) Free vibration.

Table S1. The detailed dimension parameters of the top blade for different groups.

Group	The length of cantilever beam (L_p, mm)	The width of cantilever beam (W_p, mm)	The height of acrylic substrate (H_a, mm)
1	3	50	3
2	4	50	3
3	5	50	3
4	5	50	2
5	5	50	1
6	5	40	3
7	5	30	3

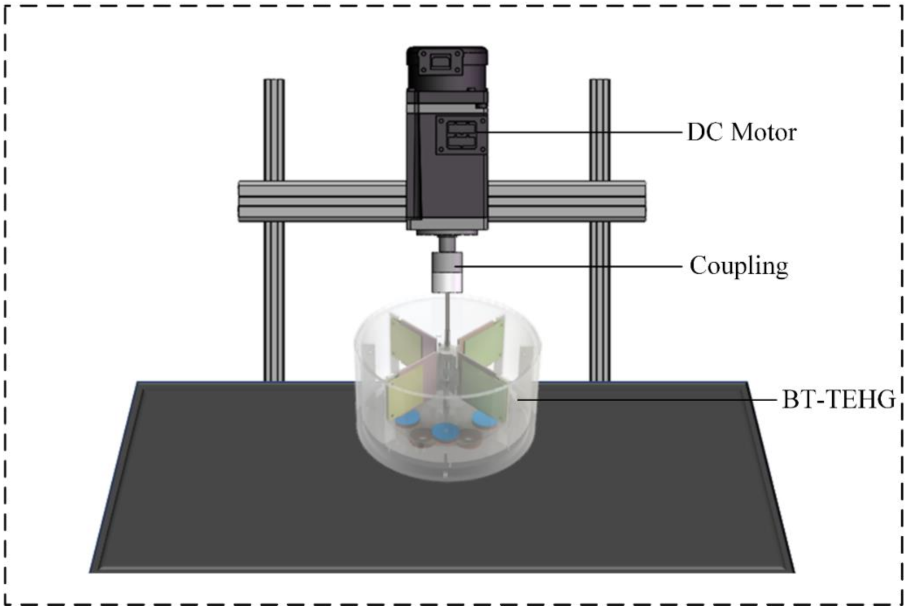

Figure S2. Schematic diagram of the motor test platform.

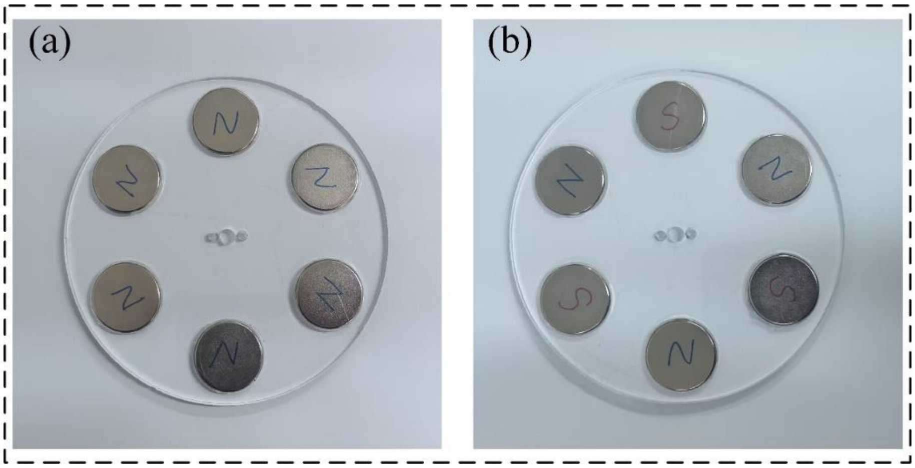

Figure S3. Arrangement of magnets: (a) Same magnetization direction of adjacent magnets. (b) Different magnetization directions of adjacent magnets.

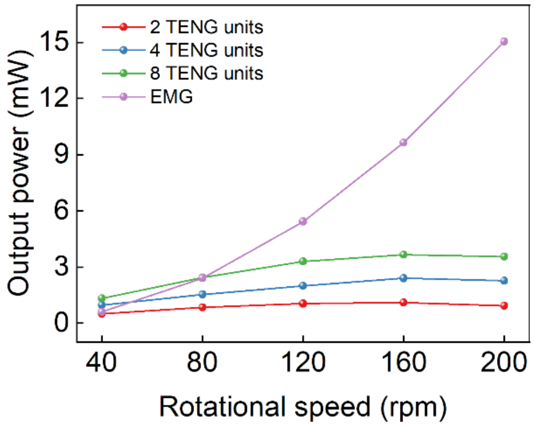

Figure S4. The output power of the standalone TENG and standalone EMG under different rotational speeds when connected to the matched resistances.

Table S2. The related parameters for energy conversion efficiency.

Wind speed (m/s)	$E_{\text{out-TENG}}$ (mJ)	$E_{\text{in-TENG}}$ (mJ)	η_{TENG} (%)	$P_{\text{out-EMG}}$ (mW)	$P_{\text{in-EMG}}$ (mW)	η_{EMG} (%)
2.2	1.436364	6.242582	23.00913	0.51	101.944682	0.500271
4	1.4	6.178007	22.66103	1.91	227.451308	0.83974
6	1.25625	6.164213	20.37973	5.45	321.960887	1.692752
8	1.2	6.197344	19.36313	9.48	363.58699	2.607354
10	0.911429	6.129338	14.86993	15.82	416.732265	3.796202

Note S1. Calculation of energy conversion efficiency

For the TENG, energy conversion efficiency η_{TENG} is defined as the ratio of output electrical energy $E_{\text{out-TENG}}$ to the input mechanical energy $E_{\text{in-TENG}}$, which can be expressed as

$$\eta_{\text{TENG}} = \frac{E_{\text{out-TENG}}}{E_{\text{in-TENG}}} \times 100\%$$

where $E_{\text{out-TENG}}$ is determined by the output power per rotational cycle of the shaft and $E_{\text{in-TENG}}$ is calculated from the elastic potential energy stored in cantilever beams when the top blades reach the maximum displacement $z_{\max}$. $E_{\text{in-TENG}}$ can be calculated as

$$E_{\text{in-TENG}} = \frac{1}{2} K_{\text{p}} dz^2 = \frac{K_{\text{p}} R_1^2 z_{\max}^2}{2r^2}$$

where dz is the tip displacement of cantilever beam corresponding to $z_{\max}$ and r is the distance between laser point and the fixed end of cantilever beam, as shown in Figure S5. Based on Hooke's law, the equivalent stiffness K_{p} (278.26 N/m) is calculated by the elastic restoring force divided by tip displacement of cantilever beam.

For the EMG, energy conversion efficiency η_{EMG} is defined as the ratio of output power $P_{\text{out-EMG}}$ to the input mechanical power $P_{\text{in-EMG}}$, which is given by

$$\eta_{\text{EMG}} = \frac{P_{\text{out-EMG}}}{P_{\text{in-EMG}}} \times 100\%$$

where $P_{\text{in-EMG}}$ is calculated from the torque T multiplied by the angular velocity ω. T is the difference in torques under conditions connected to load resistance and open circuit.

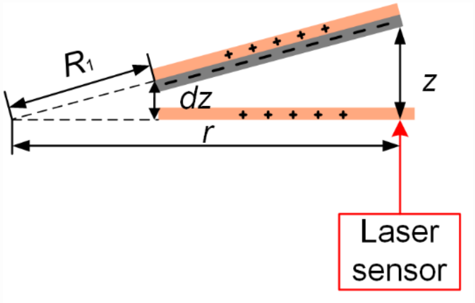

Figure S5. Schematic diagram of the tip displacement measurement of cantilever beam.
